# Trichomonas vaginalis Detection in Urogenital Specimens from Symptomatic and Asymptomatic Men and Women by Use of the cobas TV/MG Test

**DOI:** 10.1128/JCM.00264-21

**Published:** 2021-09-20

**Authors:** Barbara Van Der Pol, Arundhati Rao, Melinda B. Nye, Steven Chavoustie, Aaron Ermel, Clair Kaplan, David Eisenberg, Philip A. Chan, Leandro Mena, Sixto Pacheco, Ken B. Waites, Li Xiao, Smitha Krishnamurthy, Ruchika Mohan, Rasa Bertuzis, Chris L. McGowin, Rodney Arcenas, Elizabeth M. Marlowe, Stephanie N. Taylor

**Affiliations:** a School of Medicine, University of Alabama at Birmingham, Birmingham, Alabama, USA; b Baylor Scott & White Health, Temple, Texas, USA; c Laboratory Corporation of America Holdings, Burlington, North Carolina, USA; d Segal Trials, Inc., Miami Lakes, Florida, USA; e Indiana University School of Medicine, Indianapolis, Indiana, USA; f Planned Parenthood of Southern New England, New Haven, Connecticut, USA; g School of Medicine, Washington University in St. Louis, St. Louis, Missouri, USA; h Brown University, Providence, Rhode Island, USA; i The University of Mississippi Medical Center, Jackson, Mississippi, USA; j BioCollections Worldwide, Inc., Miami, Florida, USA; k Roche Molecular Systems Inc., Pleasanton, California, USA; l Quest Diagnostics Infectious Disease, San Juan Capistrano, California, USA; m Louisiana State University Health Sciences Center, New Orleans, Louisiana, USA; Mayo Clinic

**Keywords:** NAAT, *Trichomonas vaginalis*, molecular methods, urogenital

## Abstract

Trichomonas vaginalis is a prevalent sexually transmitted infection (STI). Diagnosis has historically relied on either microscopic analysis or culture, the latter being the previous gold standard. However, these tests are not readily available for male diagnosis, generally only perform well for symptomatic women, and are not as sensitive as nucleic acid amplification tests (NAATs). Men are largely asymptomatic but carry the organism and transmit to their sexual partners. This multicenter, prospective study evaluated the performance of the cobas T. vaginalis/Mycoplasma genitalium (TV/MG) assay for detection of T. vaginalis DNA compared with patient infection status (PIS) defined by a combination of commercially available NAATs and culture using urogenital specimens. A total of 2,064 subjects (984 men and 1,080 women, 940 [45.5%] symptomatic, 1,124 [54.5%] asymptomatic) were evaluable. In women, sensitivity ranged from 99.4% (95% confidence interval [CI] 96.8 to 99.9%) using vaginal samples to 94.7% (95% CI 90.2 to 97.2%) in PreservCyt samples. Specificity ranged from 98.9 to 96.8% (95% CI 95.4 to 97.8%). In men, the cobas TV/MG assay was 100% sensitive for the detection of T. vaginalis in both male urine samples and meatal swabs, with specificity of 98.4% in urine samples and 92.5% in meatal swabs. The cobas TV/MG is a suitable diagnostic test for the detection of T. vaginalis, which could support public health efforts toward infection control and complement existing STI programs.

## INTRODUCTION

Trichomonas vaginalis is considered one of the most common curable sexually transmitted infections (STIs) (1, 2), with the World Health Organization (WHO) estimating 156 million cases in 2016, a higher prevalence than Chlamydia trachomatis, Neisseria gonorrhoeae, or syphilis (3). T. vaginalis is currently not a reportable disease and the true estimation of disease prevalence is not currently known. Some of the factors contributing to this are a lack of routine testing and nonspecific symptomatology, and infected men being predominantly asymptomatic (4, 5).

A large proportion of T. vaginalis infections are asymptomatic; however, symptoms can include urethral discharge, primarily in males, and abnormal vaginal discharge, dysuria, itching, irritation, and abdominal pain in females (1). The consequences of untreated T. vaginalis infection may include pelvic inflammatory disease (PID) and adverse outcomes of pregnancy (6). T. vaginalis infection has also been shown to increase the risk of HIV by 50% via several mechanisms, including damage to the vaginal epithelial membrane by the protozoa (6).

The Centers for Disease Control and Prevention (CDC) recommends that women presenting with symptoms are tested for T. vaginalis, but do not recommend generalized screening of asymptomatic women (7). However, screening is recommended for women living in areas with higher than average prevalence or those who report behaviors that may have resulted in exposure to STIs, or who are HIV positive (8). Coinfection of T. vaginalis with C. trachomatis and/or N. gonorrhoeae in women has been previously reported (9) and symptoms can overlap between infections. Therefore, T. vaginalis infections may be missed and left untreated if C. trachomatis and/or N. gonorrhoeae positivity is presumed to be the cause. In populations with high levels of STI exposure, the inclusion of T. vaginalis testing alongside C. trachomatis/N. gonorrhoeae testing is likely to dramatically increase case finding (10). A study by Sena et al. looked at the prevalence of T. vaginalis infections in the male partners of women with trichomoniasis and observed that 71.7% of the men were also infected, of which 77.3% were asymptomatic (11). Men are seldom tested for T. vaginalis at the time of testing for C. trachomatis/N. gonorrhoeae due to a lack of recommendations and testing methodologies, which is further complicated by the largely asymptomatic nature of T. vaginalis infections. Reliable testing platforms and more data are needed to understand the true prevalence of symptomatic and asymptomatic infections, particularly in male populations.

Laboratory diagnosis of T. vaginalis previously relied on either microscopic analysis of a saline wet mount prepared from the female patient’s discharge, examination of spun sediment in male urine (rarely available in outpatient settings), or culture, the latter formerly being the gold standard (7, 12). These tests are highly specific, although they generally only perform well for symptomatic women and, even then, they are not as sensitive as nucleic acid amplification tests (NAATs) (7, 12). NAATs for T. vaginalis are available, such as the Aptima CV/TV assay (Hologic, San Diego, USA), the cobas TV/MG assay (Roche Diagnostics, Pleasanton, USA), Xpert TV (Cepheid, Sunnydale, USA), and Amplivue Trichomonas Assay (Quidel Corporation, Athens, USA). There are limited studies available on the performance of these assays; however, they generally demonstrate more than 96% sensitivity for the detection of T. vaginalis (13–15).

The objective of this study was to evaluate the performance of a new NAAT, the cobas TV/MG assay performed on the cobas 6800/8800 systems, for the detection of T. vaginalis in symptomatic and asymptomatic male and female urogenital samples, compared to a prespecified patient infection status (PIS). The PIS was defined using a combination of a Food and Drug Administration (FDA)-cleared NAAT and T. vaginalis culture.

## MATERIALS AND METHODS

### Patient population and ethics.

This multicenter, prospective clinical study recruited participants at nine sites in the USA: Birmingham, AL; Indianapolis, IN; Jackson, MS; Miami, FL; New Haven, CT; New Orleans, LA; Oakland, CA; Providence, RI; and St. Louis, MO. Male and female patients, whether symptomatic or asymptomatic, were eligible if they were (i) aged ≥14 years, (ii) reported sexual activity within the past 6 months, and (iii) were attending family planning, obstetrics and gynecology, or STI clinics.

Patients were ineligible if they had (i) previously enrolled in the study; (ii) used antimicrobial agents active against T. vaginalis (metronidazole or tinidazole) within the 21 days prior to sample collection; (iii) used Replens (Church & Dwight, Co., Inc., Princeton, NJ), RepHresh Odor Eliminating Vaginal Gel (Church & Dwight, Co., Inc., Princeton, NJ), RepHresh Clean and Balance (Church & Dwight, Co., Inc., Princeton, NJ) or products containing metronidazole within 21 days prior to specimen collection; (iv) had undergone a total hysterectomy; or (v) had a contraindication to the Papanicolaou test or cervical sampling.

Participants were classified as symptomatic for T. vaginalis infection if they reported any of the following: dysuria; coital issues (pain, difficulty, or bleeding); pelvic pain; abnormal vaginal discharge; unusual vaginal odor; pelvic, uterine or ovarian pain; penile discharge; testicular pain; scrotal pain; or swelling, itching, burning, redness, or soreness of the genitals. This study was conducted in compliance with the International Conference on Harmonisation (ICH) Good Clinical Practice (GCP) Guidelines and the US FDA regulations. The study protocol was submitted to an Institutional Review Board (IRB) to ensure the local and FDA requirements were met prior to the start of the study. All participants were required to provide informed signed consent. This manuscript was prepared in accordance with the STARD guidelines for reporting of clinical studies (16).

### Specimen collection.

Women provided specimens in the following order: a first catch urine (FCU); vaginal swabs; an endocervical swab in cobas PCR medium; and a cervical specimen in PreservCyt solution obtained with a spatula, cytobrush, or broom. Participants were randomized to either the self-obtained or the clinician-obtained arm for collection of vaginal swabs used in the cobas assay. Participants within the self-collected arm obtained their self-collected vaginal swab first, and the remaining swabs were clinician-collected. In the clinician-collected arm, all vaginal swabs were clinician collected. The T. vaginalis swabs were collected as follows; Hologic APTIMA TV assay, the cobas TV assay, and finally the InPouch TV assay (Biomed Diagnostics, White City, OR) specimen, which was collected last in the series due to the use of a speculum. Following collection, the clinician transferred the swabs to the relevant transport medium, as per the respective comparator test’s standard procedure. Participants randomized to the clinician-collected arm had their vaginal swab specimen for cobas testing collected by the clinician. Both the endocervical swab and the liquid-based cytology (LBC) sample were collected for assessment with the cobas assay only.

Men first provided self- or clinician-collected meatal swabs (collected in randomized order) for use with the cobas test, followed by an FCU sample. The FCU sample was aliquoted into the manufacturer’s collection device for the test assays as per the instructions for use (the penile meatal swab is not an FDA-cleared sample type for the detection of TV in the cobas TV/MG assay for use on the cobas 6800/8800 systems).

### Sample testing.

Testing with the cobas TV/MG was performed at three sites using at least three lots of investigational reagents. All cobas specimens from a single subject were tested at one individual test site. Specimens from runs with control/operator failures were retested if sufficient sample remained, and individual invalid results were also repeated if sample volume allowed. If invalid results remained invalid upon retesting and there was insufficient volume for further testing, the result remained invalid.

Female and male urogenital specimens were assessed by NAAT using the APTIMA TV assay and the Cepheid Xpert TV assay (Cepheid, Sunnyvale, CA, USA), respectively, and the InPouch TV Culture System. For female samples, the PIS was deemed positive if their vaginal samples tested positive by either the APTIMA TV assay or the InPouch TV Culture System tests, in accordance with FDA guidelines (17) at the time of the study inception. A negative culture result, plus an invalid NAAT result, was deemed as indeterminate. For male samples, the PIS was deemed positive if male urine samples tested positive via either the Cepheid Xpert TV assay or the InPouch TV Culture System. Again, where there was a negative result and an invalid result, the PIS was deemed indeterminate.

### Data analysis.

Test results for each assay were interpreted according to the instructions for use. All data analyses were performed using SAS/STAT software (version 9.4) (SAS Institute Inc., Cary, NC, USA) and in accordance with FDA guidance (18). The clinical performance of the cobas test for the detection of T. vaginalis was evaluated by comparing test results for each sample type to the PIS. The sensitivity, specificity, positive predictive value (PPV), and negative predictive value (NPV) were calculated by sex, specimen type, and symptom status and compared to an infected status algorithm for each sex. In the algorithm, the designation of a subject as infected or noninfected was based on the combination of results obtained from comparator assays. Additionally, results were analyzed separately for self-collected and clinician-collected vaginal and penile meatal swab specimens and by testing sites. The two-sided 95% confidence intervals (CIs) for the estimates of sensitivity, specificity, PPV, and NPV were estimated using the Score method (19).

## RESULTS

### Patient characteristics.

A total of 2,064 subjects were evaluable for T. vaginalis, including 984 men and 1,080 women (Table 1). Twenty-six samples were excluded from T. vaginalis analyses for invalid results, protocol deviation, or insufficient sample volume: 3 female urine, 1 clinician-collected vaginal swab sample, 1 self-collected vaginal swab sample, 6 PreservCyt samples, 6 endocervical swab samples, 1 male urine sample, 2 male clinician-collected samples, 2 male self-collected samples, 1 vaginal swab without collection method information (self or clinician) and 3 meatal swabs without collection method information (self or clinician). Of the women in the final analysis, 542 were included in the clinician-collected and 535 were included in the self-collected arm of the study, with 1,074 women providing valid samples for PreservCyt and endocervical swab analysis and 1,077 women providing valid urine samples. Of the men, 488 were included in the clinician-collected meatal arm and 489 were included in the self-collected meatal arm of the study. A total of 983 men provided valid urine samples for analysis. Positivity for T. vaginalis in this study, based on the PIS, was 15.8% (171/1,080) among all women, and 2.3% (23/983) among all men evaluated (Table 2). The prevalence of T. vaginalis in asymptomatic and symptomatic women was 12.1% (55/455) and 18.6% (11/625), respectively. The prevalence of T. vaginalis in asymptomatic and symptomatic men was 1.5% (10/668) and 4.1% (13/315), respectively. Additional information regarding male and female T. vaginalis positivity by state location can be found in Table S1 in the supplemental information.

**TABLE 1:** Baseline demographics and characteristics

Characteristic^*a*^	Value(s)
Total (*n*)	2,064
Age, yrs (mean ± SD)	35.5 ± 12.5
Male (*n* [%]) Female (*n* [%])	984 (47.7) 1,080 (52.3)
American Indian/Alaskan Native (*n* [%]) Asian (*n* [%]) Black/African American (*n* [%]) Native Hawaiian/Pacific Islander (*n* [%]) White (*n* [%]) Multiple/other (*n* [%]) Not reported (*n* [%])	3 (0.1) 13 (0.6) 1,433 (69.4) 5 (0.2) 536 (26.0) 54 (2.6) 20 (1.0)
Symptomatic (*n* [%]) Asymptomatic (*n* [%])	940 (45.5) 1,124 (54.5)
Pregnant (female only (*n* [%]))	3 (0.3)
Family planning clinic (*n* [%]) Obstetrics/gynecology clinic (*n* [%]) STI clinic (*n* [%]) Family planning/STI clinic (*n* [%])	521 (25.2) 260 (12.6) 741 (35.9) 542 (26.3)

a *n*, number of samples; SD, standard deviation; STI, sexually transmitted infection.

**TABLE 2:** Clinical performance compared with PIS by gender, sample type, and symptom status^*a*^

Sample type	Total (*N*)	% Prevalence (*n*/*N*)	% Sensitivity (*n*/*N*)	95% CI	% Specificity (*n*/*N*)	95% CI
Female participants
Urine
Symptomatic Asymptomatic Overall	622 455 1,077	18.6 (116/622) 12.1 (55/455) 15.9 (171/1077)	97.4 (113/116) 98.2 (54/55) 97.7 (167/171)	92.7–99.1 90.4–99.7 94.1–99.1	98.8 (500/506) 98.5 (394/400) 98.7 (894/906)	97.4–99.5 96.8–99.3 97.7–99.2
Vaginal swab (both clinician- and self-collected)
Symptomatic Asymptomatic Overall	623 454 1,077	18.6 (116/623) 12.1 (55/454) 15.9 (171/1077)	100 (116/116) 98.2 (54/55) 99.4 (170/171)	96.8–100 90.4–99.7 96.8–99.9	97.0 (492/507) 96.5 (385/399) 96.8 (877/906)	95.2–98.2 94.2–97.9 95.4–97.8
PreservCyt samples
Symptomatic Asymptomatic Overall	622 452 1,074	18.5 (115/622) 12.2 (55/452) 15.8 (170/1074)	93.9 (108/115) 96.4 (53/55) 94.7 (161/170)	88.0–97.0 87.7–99.0 90.2–97.2	99.2 (503/507) 98.5 (391/397) 98.9 (894/904)	98.0–99.7 96.7–99.3 98.0–99.4
Endocervical swab
Symptomatic Asymptomatic Overall	620 454 1,074	18.5 (115/620) 12.1 (55/454) 15.8 (170/1074)	97.4 (112/115) 98.2 (54/55) 97.6 (116/170)	92.6–99.1 90.4–99.7 94.1–99.1	98.8 (499/505) 97.2 (388/399) 98.1 (887/904)	97.4–99.5 95.1–98.5 97.0–98.8
All female subjects^*b*^
Symptomatic Asymptomatic Overall	625 455 1,080	18.6 (116/625) 12.1 (55/455) 15.8 (171/1080)				
Male participants
Urine
Symptomatic Asymptomatic Overall	315 668 983	4.1 (13/315) 1.5 (10/668) 2.3 (23/983)	100 (13/13) 100 (10/10) 100 (23/23)	77.2–100 72.2–100 85.7–100	98.3 (297/302) 98.5 (648/658) 98.4 (945/960)	96.2–99.3 97.2–99.2 97.4–99.1
Meatal swab (both clinician- and self-collected)
Symptomatic Asymptomatic Overall	315 662 977	4.1 (13/315) 1.7 (11/662) 2.5 (24/977)	100 (13/13) 100 (11/11) 100 (24/24)	77.2–100 74.1–100 86.2–100	91.1 (275/302) 93.2 (607/651) 92.5 (882/953)	87.3–93.8 91.0–94.9 90.7–94.1
All male subjects^*b*^
Symptomatic Asymptomatic Overall	315 668 983	4.1 (13/315) 1.5 (10/668) 2.3 (23/983)				

a CI, confidence interval; *N*, total number of samples; *n*, number of *T. vaginalis*-positive samples (for prevalence), number of positive samples with accurate result (for sensitivity), or number of negative samples with accurate result (for specificity); PIS, patient infected status.

b These numbers represent the overall prevalence of TV infection in male and female subjects.

### Assay performance for the detection of T. vaginalis.

For female specimens (asymptomatic and symptomatic) the sensitivity ranged from 99.4% (95% CI 96.8 to 99.9%) for vaginal samples to 94.7% (95% CI 94.1 to 99.1%) for LBC samples in PreservCyt (Table 2). Specificity across the female specimen types was >96.8%. The cobas TV/MG assay was 100% sensitive for the detection of T. vaginalis in both male urine samples and meatal swabs. Specificity of the cobas assay was higher in male urine samples (98.4%) compared with meatal swabs (92.5%).

Compared with patient infection status (PIS), both clinician-collected and self-collected male and female samples had similar performance with the cobas TV assay (Table 3). Self-collected vaginal swab samples had slightly higher sensitivity and specificity versus clinician-collected samples; however, the differences were not significant (*P* = 1.00 and *P* = 0.21 for sensitivity and specificity, respectively). Self-collected meatal swab samples showed similar sensitivity (both 100%) and specificity (92.5% versus 92.6%) performance to clinician-collected samples (*P* = 1.00 for both sensitivity and specificity, respectively). In female samples, the PPV of the cobas assay for T. vaginalis across different clinics and sample types was 50.0 to 100% and the NPV was 97.3 to 100% (Table S2). In male samples, the PPV of the cobas assay for T. vaginalis was 13.3 to 100% and the NPV was 100% (Table S2).

**TABLE 3:** Clinical performance of self-collected versus clinician collected vaginal/meatal swab samples^*c*^

Sample type	Total (*N*)	% Sensitivity (*n*/*N*)	95% CI	% Specificity (*n*/*N*)	95% CI
Female vaginal swab samples^*a*^					
Clinician-collected					
Symptomatic Asymptomatic Overall	335 207 542	100 (71/71) 96.4 (27/28) 99.0 (98/99)	94.9–100 82.3–99.4 94.5–99.8	96.6 (255/264) 95.0 (170/179) 95.9 (425/443)	93.6–98.2 90.7–97.3 93.7–97.4
Self-collected					
Symptomatic Asymptomatic Overall	288 247 535	100 (45/45) 100 (27/27) 100 (72/72)	92.1–100 87.5–100 94.9–100	97.5 (237/243) 97.7 (215/220) 97.6 (452/463)	94.7–98.9 94.8–99.0 95.8–98.7
Male meatal swab samples^*b*^					
Clinician-collected					
Symptomatic Asymptomatic Overall	169 319 488	100 (7/7) 100 (7/7) 100 (14/14)	64.6–100 64.6–100 78.5–100	91.4 (148/162) 93.3 (291/312) 92.6 (439/474)	86.0–94.8 89.9–95.6 89.9–94.6
Self-collected					
Symptomatic Asymptomatic Overall	146 343 489	100 (6/6) 100 (4/4) 100 (10/10)	61.0–100 51.0–100 72.2–100	90.7 (127/140) 93.2 (316/339) 92.5 (443/479)	84.8–94.5 90.0–95.4 89.8–94.5

a Overall difference in sensitivity (95% CI) and specificity (95% CI) for clinician-collected versus self-collected vaginal swabs was −1.0 (−3.0, 1.0), *P* = 1.00; and −1.7% (−4.0%, 0.6%), *P* = 0.21, respectively.

b Overall difference in sensitivity (95% CI) and specificity (95% CI) for clinician-collected versus self-collected meatal swabs was 0% (−23.2%, 30.9%), *P* = 1.00; and 0.1% (−3.2%, 3.5%), *P* = 1.00, respectively.

c CI, confidence interval; *N*, total number of samples; *n*, number of positive samples with accurate result (for sensitivity) or number of negative samples with accurate result (for specificity).

**FIG 1:**
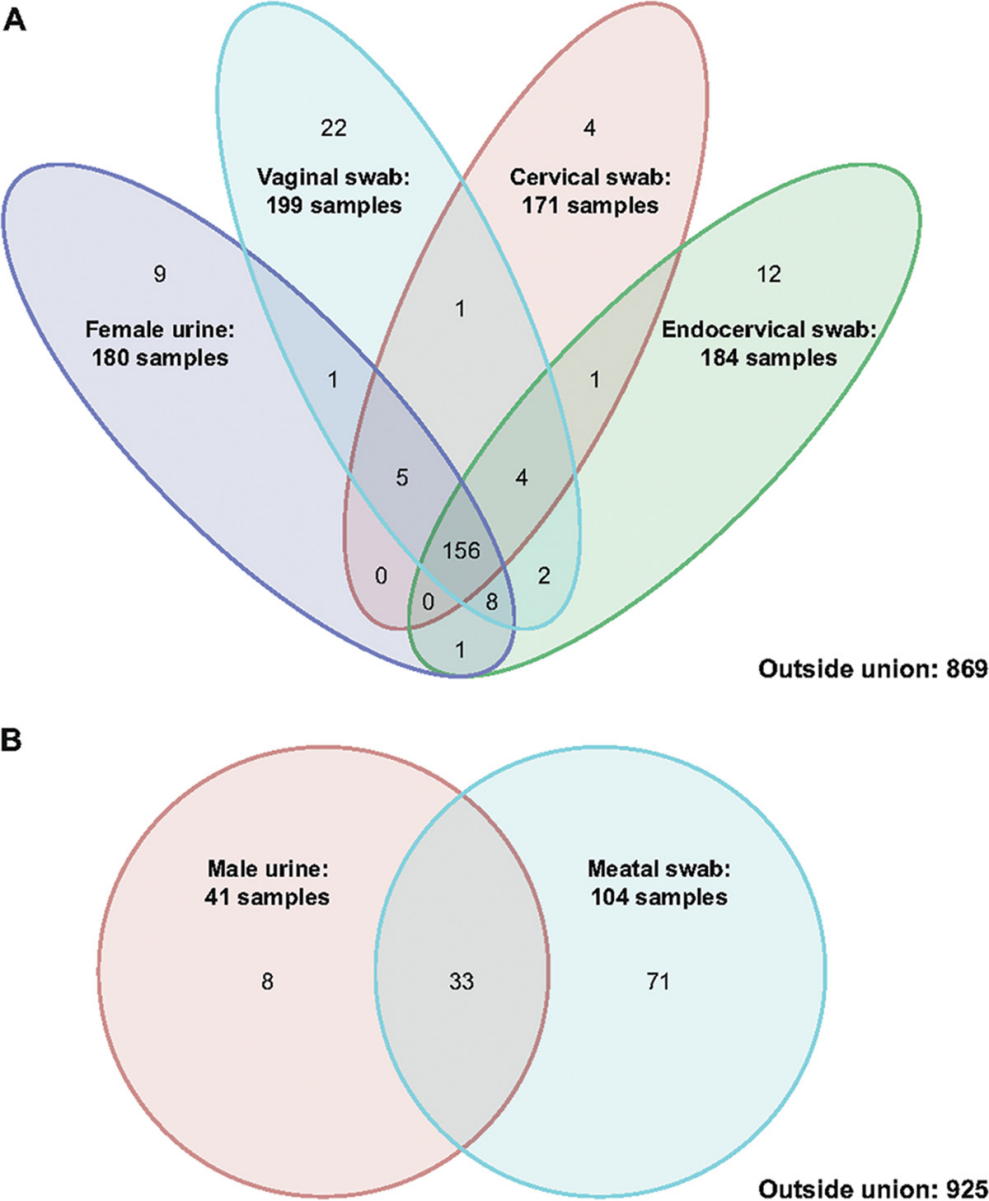
Venn diagrams comparing the cobas T. vaginalis positives in female urogenital samples (A) and male urogenital samples (B).

**FIG 2:**
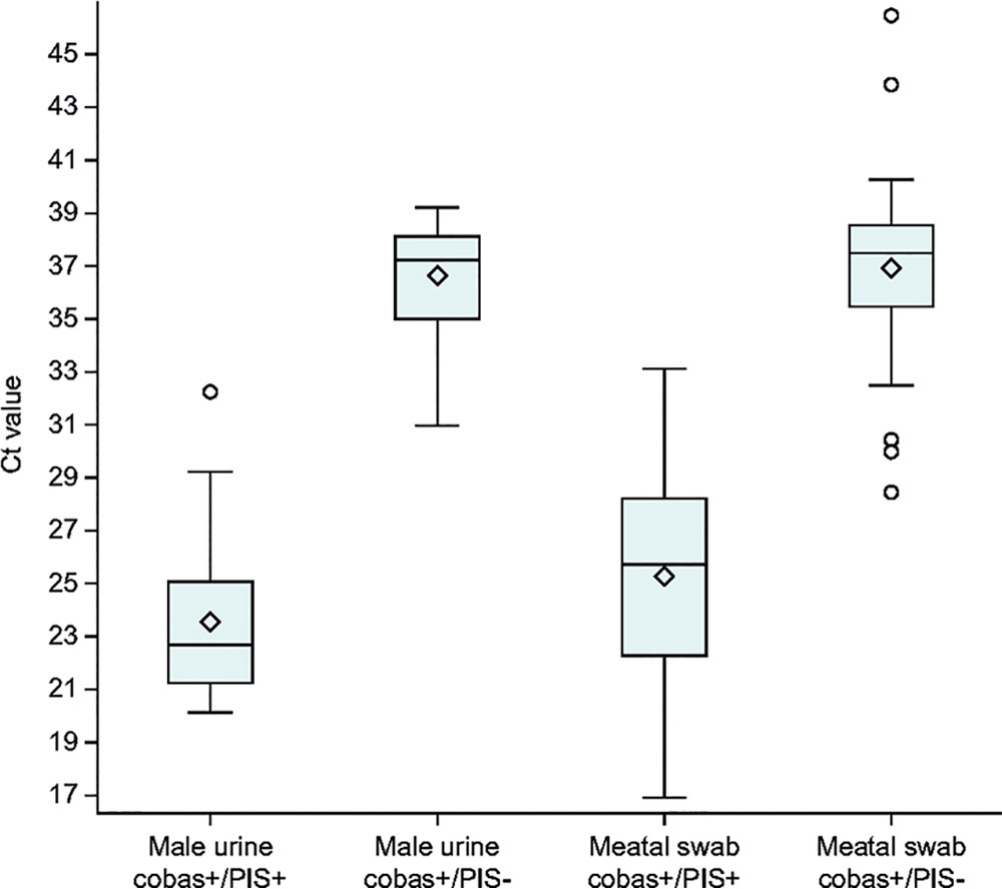
Distribution of *C_T_* values from T. vaginalis positive results with the cobas TV/MG assay in evaluable males. The diamonds represent the mean values and the lines inside the boxes represent the median values. The error bars represent standard deviation, with the circles representing individual samples outside of the error bars. *C_T_*, cycle threshold; PIS, patient infection status.

Figure 1 illustrates cobas T. vaginalis positivity across all samples, regardless of infection status. For female samples, most positive samples were positive across all sample types (Fig. 1A). In Fig. 1B, the data show 71/104 meatal swab-only positives. For male urine, only 23/41 positives were confirmed by PIS. However, the cobas meatal swab and urine results were both positive for 10 patients not identified as such by the PIS. On average, both male urine and meatal swab samples that were positive by cobas and negative by PIS had higher cycle threshold (*C_T_*) values compared with those samples positive by both cobas and PIS (Fig. 2). The *C_T_* values for male urine and meatal swab samples that were positive by the cobas assay can be found in Table S3.

## DISCUSSION

In this study, the cobas assay was highly sensitive and specific for the detection of T. vaginalis in both male and female urogenital samples, with sensitivities greater than or similar to those seen with other NAATs for T. vaginalis (7, 9, 12, 20, 21). The sensitivity and specificity of cobas, when compared to the PIS, showed that male urine samples and female vaginal swab samples are preferred for detection of T. vaginalis infection. It is interesting to observe that regardless of the PIS, cobas TV-positive results for men show a higher detection rate in penile meatal swabs (104) versus urine (41), and only 33 men had T. vaginalis detected in both their urine and meatal swab samples (Fig. 1B). A previous study observed that among the paired specimens of meatal swabs and urine, T. vaginalis was detected in a higher percentage of meatal swabs compared with urine samples (80.4% versus 39.3%) (22). Similar to our clinical trial study, Dize et al. collected the meatal swabs prior to the collection of urine (22). Another study also observed a higher detection rate among the paired collections of meatal swabs (8.0%) versus urine (1.7%) (23). It is important to note that in this particular study, Chernesky et al. randomized the sequence of collection between these two specimens (23) and therefore this mitigates the concern that the meatal swab collected the majority of T. vaginalis if it was collected prior to the urine collection. This would suggest that the higher detection rates in meatal swabs may be inherently due to the parasite’s pathophysiology; T. vaginalis might adhere to the urethra and be less likely to detach in urine versus other samples (24). The higher *C_T_* values for male samples positive by cobas and negative by PIS compared with the lower *C_T_* values for samples positive by both cobas and PIS may also indicate the increased sensitivity of cobas for detecting samples with lower parasite load compared with the previous methods used to define PIS (Fig. 2). Ultimately, the clinical significance of meatal swabs as a viable alternative sample type compared to urine remains to be determined.

Although there are no official guidelines or recommendations for T. vaginalis testing in men, if testing becomes readily available then those guidelines may change, and male partners of females diagnosed with T. vaginalis infection can be offered testing with urine or urethral swabs to confirm diagnosis prior to treatment (25). The clinical significance and utility of the penile meatal swab is currently unknown, although this sample has been shown to have potential utility in a number of studies for the detection of T. vaginalis and other STI pathogens (C. trachomatis, *N. gonorrhea*, and M. genitalium) (22, 23, 26). The collection process for penile meatal swabs may also be more comfortable for patients compared with urethral swab collection.

The high performance of the vaginal swab samples collected in cobas PCR medium indicate that this sampling method is the optimal specimen type for use with the cobas CT/NG assay, which should facilitate ease of testing during a patient clinic visit (27). Only one sample tested positive with the APTIMA TV alone, in an asymptomatic female patient, who would have been defined by PIS as infected.

As noted, both the clinician-collected and self-collected male meatal and female vaginal swab samples were highly sensitive and specific. This is very important, as self-testing can increase the likelihood that patients will access testing and, in light of the current COVID-19 pandemic and rising incidence of STIs, it may be increasingly important in enabling patients to access medical care and diagnosis (28).

The prevalence of T. vaginalis in this study, at 15.8% in female subjects and 2.3% in male subjects, was higher than some previous estimates of T. vaginalis prevalence, particularly in asymptomatic female subjects, which was 12.1% in this study (7, 9, 12). The prevalence of T. vaginalis in male subjects was low, as was expected based on data in the literature (7, 12, 26), and may have been underestimated due to the collection of meatal swabs prior to urine specimens which were used to define infection status. If one assumes that those meatal swabs that gave positive results when the cobas urine result was also positive, but where PIS was negative, were in fact true positives, then positivity would have been 4.2% (41/983). Furthermore, if the 71 meatal samples that were not confirmed with other urine tests were compared head to head with meatal samples using a comparator NAAT (which is a study limitation due to the lack of another FDA-cleared NAAT for the detection of T. vaginalis in men) the positivity would be estimated to be 11.5% (113/983).

In conclusion, the high performance of the cobas TV/MG assay for use on the cobas 6800/8800 systems, in both clinician- and self-collected urogenital samples from symptomatic and asymptomatic men and women, means the cobas assay can reliably detect the presence and absence of T. vaginalis in urogenital samples and is a suitable clinical test for the diagnosis of T. vaginalis. The cobas 6800/8800 offers an optimal systems approach for use alongside other commercially available STI tests. The cobas TV/MG assay fulfils an unmet medical need for the testing of patients for diagnosis, supports public health efforts toward the control of T. vaginalis, and complements existing STI programs.
