# Supplementary material for: Trichomonas vaginalis Detection in Urogenital Specimens from Symptomatic and Asymptomatic Men and Women by Use of the cobas TV/MG Test
Source: J Clin Microbiol. 2021 Sep 20;59(10):e00264-21. doi: 10.1128/JCM.00264-21 (PMC8451429; doi:10.1128/JCM.00264-21)
Supplement: Supplemental file 1 — Tables S1 to S3. Download JCM.00264-21-s0001.pdf, PDF file, 0.06 MB [file jcm.00264-21-s0001.pdf]

Supplementary Table 1. Numbers of patients enrolled by clinic, gender, and *T. vaginalis* positivity.

Clinic (by State of location)	Men		Women	
	Total enrolled	Total positive for *T. vaginalis*	Total enrolled	Total positive for *T. vaginalis*
Alabama	72	1 (1.4%)	1126	147 (13.1%)
California	300	13 (4.3%)	597	159 (26.6%)
Connecticut	392	2 (0.5%)	593	37 (6.2%)
Florida (site 1)	300	13 (4.3%)	600	156 (26.2%)
Florida (site 2)	285	4 (1.4%)	575	136 (23.7%)
Indiana	74	2 (2.7%)	47	5 (10.6%)
Louisiana	387	5 (1.3%)	340	52 (15.3%)
Missouri	82	–	591	47 (8.0%)
Mississippi	96	1 (1.0%)	–	–
Rhode Island	142	–	–	–

Data shown are cobas positive results, regardless of the PIS.

Supplementary Table 2. Clinical performance compared with PIS by gender, sample type, and clinic.

Sample type[a]	State	Total (N)	Sensitivity % (N/N)	95% score CI	Specificity % (N/N)	95% score CI	Prevalence (%)	PPV (%)	NPV (%)
Female									
Urine	Alabama	274	100.0 (35/35)	(90.1, 100.0)	99.2 (237/239)	(97.0, 99.8)	12.8	94.6	100.0
	California	135	94.6 (35/37)	(82.3, 98.5)	96.9 (95/98)	(91.4, 99.0)	27.4	92.1	97.9
	Connecticut	149	100.0 (9/9)	(70.1, 100.0)	99.3 (139/140)	(96.1, 99.9)	6.0	90.0	100.0
	Florida	149	97.4 (37/38)	(86.5, 99.5)	96.4 (107/111)	(91.1, 98.6)	25.5	90.2	99.1
	Florida (site 2)	133	100.0 (29/29)	(88.3, 100.0)	99.0 (103/104)	(94.8, 99.8)	21.8	96.7	100.0
	Indianapolis	10	100.0 (1/1)	(20.7, 100.0)	100.0 (9/9)	(70.1, 100.0)	10.0	100.0	100.0
	Louisiana	83	100.0 (12/12)	(75.8, 100.0)	98.6 (70/71)	(92.4, 99.8)	14.5	92.3	100.0
	Missouri	144	90.0 (9/10)	(59.6, 98.2)	100.0 (134/134)	(97.2, 100.0)	6.9	100.0	99.3
	Overall	1077	97.7 (167/171)	(94.1, 99.1)	98.7 (894/906)	(97.7, 99.2)	15.9	93.3	99.6
Vaginal swab (both clinician- and self-collected)	Alabama	276	100.0 (35/35)	(90.1, 100.0)	98.8 (238/241)	(96.4, 99.6)	12.7	92.1	100.0
	California	135	97.3 (36/37)	(86.2, 99.5)	92.9 (91/98)	(86.0, 96.5)	27.4	83.7	98.9
	Connecticut	148	100.0 (9/9)	(70.1, 100.0)	98.6 (137/139)	(94.9, 99.6)	6.1	81.8	100.0
	Florida	149	100.0 (38/38)	(90.8, 100.0)	96.4 (107/111)	(91.1, 98.6)	25.5	90.5	100.0
	Florida (site 2)	132	100.0 (29/29)	(88.3, 100.0)	92.2 (95/103)	(85.4, 96.0)	22.0	78.4	100.0
	Indianapolis	11	100.0 (1/1)	(20.7, 100.0)	100.0 (10/10)	(72.2, 100.0)	9.1	100.0	100.0
	Louisiana	83	100.0 (12/12)	(75.8, 100.0)	97.2 (69/71)	(90.3, 99.2)	14.5	85.7	100.0
	Missouri	143	100.0 (10/10)	(72.2, 100.0)	97.7 (130/133)	(93.6, 99.2)	7.0	76.9	100.0
	Overall	1077	99.4 (170/171)	(96.8, 99.9)	96.8 (877/906)	(95.4, 97.8)	15.9	85.4	99.9
PreservCyt samples	Alabama	275	94.3 (33/35)	(81.4, 98.4)	98.8 (237/240)	(96.4, 99.6)	12.7	91.7	99.2
	California	135	97.3 (36/37)	(86.2, 99.5)	99.0 (97/98)	(94.4, 99.8)	27.4	97.3	99.0
	Connecticut	147	88.9 (8/9)	(56.5, 98.0)	100.0 (138/138)	(97.3, 100.0)	6.1	100.0	99.3

	Florida	148	91.9 (34/37)	(78.7, 97.2)	99.1 (110/111)	(95.1, 99.8)	25.0	97.1	97.3
	Florida (site 2)	133	100.0 (29/29)	(88.3,100.0)	99.0 (103/104)	(94.8, 99.8)	21.8	96.7	100.0
	Indianapolis	11	100.0 (1/1)	(20.7, 100.0)	100.0 (10/10)	(72.2, 100.0)	9.1	100.0	100.0
	Louisiana	83	100.0 (12/12)	(75.8, 100.0)	100.0 (71/71)	(94.9, 100.0)	14.5	100.0	100.0
	Missouri	142	80.0 (8/10)	(49.0, 94.3)	97.0 (128/132)	(92.5, 98.8)	7.0	66.7	98.5
	Overall	1074	94.7 (161/170)	(90.2, 97.2)	98.9 (894/904)	(98.0, 99.4)	15.8	94.2	99.0
Endocervical swab	Alabama	274	100.0 (35/35)	(90.1, 100.0)	99.6 (238/239)	(97.7, 99.9)	12.8	97.2	100.0
	California	135	94.6 (35/37)	(82.3, 98.5)	94.9 (93/98)	(88.6, 97.8)	27.4	87.5	97.9
	Connecticut	148	88.9 (8/9)	(56.5, 98.0)	100.0 (139/139)	(97.3, 100.0)	6.1	100.0	99.3
	Florida	149	97.4 (37/38)	(86.5, 99.5)	99.1 (110/111)	(95.1, 99.8)	25.5	97.4	99.1
	Florida (site 2)	131	100.0 (28/28)	(87.9, 100.0)	95.1 (98/103)	(89.1, 97.9)	21.4	84.8	100.0
	Indianapolis	11	100.0 (1/1)	(20.7, 100.0)	90.0 (9/10)	(59.6, 98.2)	9.1	50.0	100.0
	Louisiana	83	100.0 (12/12)	(75.8, 100.0)	98.6 (70/71)	(92.4, 99.8)	14.5	92.3	100.0
	Missouri	143	100.0 (10/10)	(72.2, 100.0)	97.7 (130/133)	(93.6, 99.2)	7.0	76.9	100.0
	Overall	1074	97.6 (166/170)	(94.1, 99.1)	98.1 (887/904)	(97.0, 98.8)	15.8	90.7	99.6
Male									
Urine	Alabama	34	100.0 (1/1)	(20.7, 100.0)	100.0 (33/33)	(89.6, 100.0)	2.9	100.0	100.0
	California	120	100.0 (8/8)	(67.6, 100.0)	97.3 (109/112)	(92.4, 99.1)	6.7	72.7	100.0
	Connecticut	186	100.0 (1/1)	(20.7, 100.0)	99.5 (184/185)	(97.0, 99.9)	0.5	50.0	100.0
	Florida	138	100.0 (7/7)	(64.6, 100.0)	96.2 (126/131)	(91.4, 98.4)	5.1	58.3	100.0
	Florida (site 2)	127	100.0 (2/2)	(34.2, 100.0)	98.4 (123/125)	(94.4, 99.6)	1.6	50.0	100.0
	Indianapolis	36	-	-	94.4 (34/36)	(81.9, 98.5)	0.0	0.0	100.0
	Louisiana	188	100.0 (3/3)	(43.9, 100.0)	98.9 (183/185)	(96.1, 99.7)	1.6	60.0	100.0
	Mississippi	44	100.0 (1/1)	(20.7, 100.0)	100.0 (43/43)	(91.8, 100.0)	2.3	100.0	100.0
	Missouri	41	-	-	100.0 (41/41)	(91.4, 100.0)	0.0	-	100.0
	Rhode Island	69	-	-	100.0 (69/69)	(94.7, 100.0)	0.0	-	100.0

	Overall	983	100.0 (23/23)	(85.7, 100.0)	98.4 (945/960)	(97.4, 99.1)	2.3	60.5	100.0
Meatal swab (both clinician- and self-collected)	Alabama	34	100.0 (1/1)	(20.7, 100.0)	97.0 (32/33)	(84.7, 99.5)	2.9	50.0	100.0
	California	120	100.0 (8/8)	(67.6, 100.0)	90.2 (101/112)	(83.3, 94.4)	6.7	42.1	100.0
	Connecticut	183	100.0 (2/2)	(34.2, 100.0)	94.5 (171/181)	(90.1, 97.0)	1.1	16.7	100.0
	Florida	138	100.0 (7/7)	(64.6, 100.0)	87.8 (115/131)	(81.1, 92.3)	5.1	30.4	100.0
	Florida (site 2)	125	100.0 (2/2)	(34.2, 100.0)	89.4 (110/123)	(82.8, 93.7)	1.6	13.3	100.0
	Indiana	36	-	-	80.6 (29/36)	(65.0, 90.2)	0.0	0.0	100.0
	Louisiana	187	100.0 (3/3)	(43.9, 100.0)	94.0 (173/184)	(89.6, 96.6)	1.6	21.4	100.0
	Mississippi	44	100.0 (1/1)	(20.7, 100.0)	97.7 (42/43)	(87.9, 99.6)	2.3	50.0	100.0
	Missouri	41	-	-	97.6 (40/41)	(87.4, 99.6)	0.0	0.0	100.0
	Rhode Island	69	-	-	100.0 (69/69)	(94.7, 100.0)	0.0	-	100.0
	Overall	977	100.0 (24/24)	(86.2, 100.0)	92.5 (882/953)	(90.7, 94.1)	2.5	25.3	100.0

[a]Subjects with a designated patient infection status (Infected or Non-Infected) and a valid test result with cobas TV/MG for TV are considered evaluable and included in this summary table. Clinics UMC01 and TMH01 enrolled only male subjects.
CI, confidence interval; N, number of samples; NPV, negative predictive value, PPV = positive predictive value; PIS, patient infection status.

Supplementary Table 3. Ct values for male urine and meatal swab samples positive for *T. vaginalis* by the cobas TV/MG assay.

Sample type<br>cobas/PIS result	N	Ct values						
		Mean	SD	Minimum	Lower quartile	Median	Upper quartile	Maximum
MU<br>cobas+/PIS+	23	23.46	3.05	20.15	21.25	22.69	25.06	32.29
MU<br>cobas+/PIS-	15	36.58	2.20	30.95	34.99	37.21	38.09	39.21
MS<br>cobas+/PIS+	24	25.29	4.25	16.91	22.27	25.73	28.22	33.12
MS<br>cobas+/PIS-	71	36.93	2.83	28.47	35.47	37.50	38.50	46.50

Ct, cycle threshold; MS, meatal swab; MU, male urine; N, number of samples; PIS, patient infection status; SD, standard deviation
